# Coenzyme Q10 Phytosome Formulation Improves CoQ10 Bioavailability and Mitochondrial Functionality in Cultured Cells

**DOI:** 10.3390/antiox10060927

**Published:** 2021-06-07

**Authors:** Nicola Rizzardi, Irene Liparulo, Giorgia Antonelli, Francesca Orsini, Antonella Riva, Christian Bergamini, Romana Fato

**Affiliations:** 1 Department of Pharmacy and Biotechnology, FABIT, University of Bologna, 6, 40126 Bologna, Italy; nicola.rizzardi2@unibo.it (N.R.); irene.liparulo2@unibo.it (I.L.); giorgia.antonelli@studio.unibo.it (G.A.); romana.fato@unibo.it (R.F.); 2 Indena SpA, Viale Ortles, 20139 Milan, Italy; francesca.orsini@indena.com (F.O.); antonella.riva@indena.com (A.R.)

**Keywords:** Coenzyme Q10, phytosome, ATP, mitochondria, antioxidant, ferroptosis, Ubiqsome^®^

## Abstract

Coenzyme Q10 (CoQ10) is a lipid-soluble molecule with a dual role: it transfers electrons in the mitochondrial transport chain by promoting the transmembrane potential exploited by the ATPase to synthesize ATP and, in its reduced form, is a membrane antioxidant. Since the high CoQ10 hydrophobicity hinders its bioavailability, several formulations have been developed to facilitate its cellular uptake. In this work, we studied the bioenergetic and antioxidant effects in I407 and H9c2 cells of a CoQ10 phytosome formulation (UBIQSOME^®^, UBQ). We investigated the cellular and mitochondrial content of CoQ10 and its redox state after incubation with UBQ. We studied different bioenergetic parameters, such as oxygen consumption, ATP content and mitochondrial potential. Moreover, we evaluated the effects of CoQ10 incubation on oxidative stress, membrane lipid peroxidation and ferroptosis and highlighted the connection between the intracellular concentration of CoQ10 and its antioxidant potency. Finally, we focused on the cellular mechanism that regulates UBQ internalization. We showed that the cell lines used in this work share the same uptake mechanism for UBQ, although the intestinal cell line was less efficient. Given the limitations of an in vitro model, the latter result supports that intestinal absorption is a critical step for the oral administration of Coenzyme Q10 formulations.

## 1. Introduction

Coenzyme Q10 (CoQ10) is a lipid-soluble molecule present in all cell membrane and its primary functions are to transfer electrons in the mitochondrial respiratory chain (MRC) and to accept electrons from Complex I and Complex II to donate reducing equivalents to Complex III. Together with electrons, CoQ10 transfers protons to the intermembrane space, contributing to the establishment of the transmembrane potential, utilized by the ATPase for ATP synthesis. CoQ10 is the only lipid-soluble antioxidant synthesized by the cells; in its reduced form, (CoQH2) protects cell membranes and circulating lipoproteins from lipid peroxidation [1].

In the plasma membrane, the equilibrium between the oxidized and reduced form of CoQ10 is maintained by plasma membrane redox systems, which are particularly important under oxidative stress conditions [2,3]. Recently, Doll et al. reported that the suppression of a type of cell death linked to lipid peroxidation, named ferroptosis, is mediated by reduced CoQ10 and glutathione [4,5]. Moreover, CoQ10 is involved in the de-novo synthesis of pyrimidine, in the metabolism of fatty acids and the regeneration of vitamins C and E. In recent years, a role of CoQ10 has been described in inflammation, gene expression and membrane stability [6,7,8]. The variety of functions at the cellular level accounts for its involvement in ageing and age-related diseases, such as neurodegenerative and cardiovascular diseases [9,10,11,12]. Notably, in humans, the biosynthesis of CoQ10 gradually declines after the twenties, with a concomitant decrease in tissue concentration [13]. The deficiency of CoQ10 occurs in either a primary or a secondary form. Primary CoQ10 deficiencies, due to mutations in genes encoding enzymes of its biosynthetic pathway or being involved in the regulation of the biosynthesis, cause clinically heterogeneous diseases including encephalomyopathy, severe infantile multisystemic disease and cerebellar ataxia. Secondary deficiencies could be due to mutations in genes that are not directly involved in CoQ10 biosynthesis and may occur because of dietary insufficiency or the use of drugs such as statins [14].

Starting from these premises, the supplementation with CoQ10 to maintain adequate tissue level concentrations should be therapeutically promising. Nevertheless, despite the strong rationale the results obtained were contradictory, mainly because of the poor bioavailability of the molecule due to its high lipophilicity, light sensitivity and thermolability [15]. In order to face these problems, many formulations have been developed to increase CoQ10’s low oral bioavailability, such as the Self-Nanoemulsifying Delivery System (SNEDDS) [16], oleogels [17], micellar nanoparticles [18], liposomes [19], cyclodextrin inclusion compounds [20,21] and lipid free nanoformulations [22]. In this paper, we treated a human epithelial intestine cell line (Intestine 407, I407) and a rat cardiomyoblast cell line (H9c2) with a new food-grade delivery system of CoQ10 based on lecithin, called CoQ10 phytosome (Ubiqsome^®^, UBQ) [23,24,25]. We investigated the cellular uptake of UBQ and its mitochondrial distribution by focusing on the redox state of CoQ10 after cellular internalization. Moreover, we assessed different bioenergetics effects of UBQ treatment and its protective role against oxidative stress and ferroptosis.

## 2. Materials and Methods

### 2.1. Cell Culture and Treatment

The Intestine 407 cells (I407, embryonic epithelial cells from the human intestine) were cultivated in Roswell Park Memorial Institute (RPMI) 1640 medium, supplemented with 10% fetal bovine serum (FBS) and 1% penicillin/streptomycin. The H9c2 cells (embryonic cardiomyoblasts from rat) were cultivated in High Glucose Dulbecco’s modified Eagle’s medium (DMEM), supplemented with 10% FBS and 1% penicillin/streptomycin. All cells were grown at 37 °C in 5% CO_2_ with saturating humidity. For products treatment, unless otherwise specified, cells were grown for 24 h in complete culture medium supplemented with 100 nM of CoQ10 Phytosome^®^ (UBIQSOME^®^, UBQ), which was provided by Indena S.p.A., Milan, Italy; or with 100 nM native CoQ10, which was provided by Indena S.p.A.; or with Phytosome^®^ alone (the sunflower lecithin matrix as vehicle). Cell count was performed by using the Trypan blue exclusion method [26]; protein content was assessed by using the the Lowry method [27].

### 2.2. Coenzyme Q10 Determination

The total cellular CoQ10 content was measured by HPLC using a two-pump system equipped with a photodiode array detector (Agilent, Santa Clara, CA, USA, 1100 series) as reported in [28] with minor modifications. Briefly, CoQ was extracted from cultured cells as described by Takada et al. [29]. Then, CoQ was resuspended in 0.05% Fe^3+^ in ethanol and chromatographed on a C18 Column (Kinetex, Phenomenex, Torrance, CA, USA, 2.6 μm, 100 × 4.6 mm) using an ethanol: water (97:3, *v*/*v*) mobile phase at 0.6 mL·min^−1^ flow rate.

The CoQ peak at λ = 275 nm was identified by comparison and co-elution with a standard. The quantification of CoQ was obtained by using peak area measurement compared with standard curves and normalized on cell number. Cell Counting was performed under an inverted microscope using a Burker chamber.

The mitochondrial CoQ10 content was determined by HPLC, as stated above, in mitochondria isolated from cultured cells following Spinazzi et al. [30]. The mitochondrial CoQ10 content was normalized on mitochondrial protein content determined by the Lowry method [27].

The determination of cellular oxidized and reduced CoQ10 was performed by HPLC following [31] with modifications. Briefly, for the simultaneous determination of reduced and oxidized forms, CoQ was extracted as mentioned above. Then, a portion of the hexane solution was dried on nitrogen flux and reconstituted in acetonitrile (CAN) in the presence of 100 mg/L of butylhydroxytoluene (BHT), followed by 1 min of sonication at 4 °C and vortex mixing. The sample was transferred into a vial and immediately analyzed by HPLC as described above. Oxidized CoQ peak at λ = 275 nm and reduced CoQ peak at λ = 290 nm were identified by comparison with standards and compared with standard curves.

As a total oxidized CoQ control, a portion of the hexane solution was dried on nitrogen flux and resuspended in 0.05% Fe^3+^ in ethanol, followed by sonication (2 min) and vortex mixing (30 s). The sample was transferred into a vial and analyzed at 275 nm byHPLC. Coenzyme Q7 was used as the internal standard in all the determinations.

### 2.3. Oxygen Consumption Assay

Cellular oxygen consumption in intact cells was measured by polarography using an oxygraph chamber (Instech Mod. 203, Plymouth Meeting, PA, USA), as is reported in [28]. Briefly, cells were trypsinized and washed in NaCl 0.9%. Then, oxygen consumption rate was measured in the presence and absence of oligomycin A and carbonyl cyanide 4-(trifluoromethoxy) phenylhydrazone (FCCP) and then normalized to cells number. Cell counting was performed under an inverted microscope using a Burker chamber.

### 2.4. Intracellular NAD(P)H Determination

Intracellular NAD(P)H autofluorescence was measured in intact cells using a Jasco FP-770 spectrofluorometer (Jasco, Japan) equipped with a stirring device and thermostatic control [32]. Briefly, cells were detached by trypsinization, washed with phosphate buffer and resuspended in HBSS buffer (156 mM NaCl, 3 mM KCl, 2 mM MgSO_4_, 1.25 mM KH_2_PO_4_, 2 mM CaCl_2_, 10 mM glucose and 10 mM HEPES; pH adjusted to 7.4 with NaOH), at 30 °C, by using a 3 mL quartz cuvette. After 5 min stabilization, NAD(P)H spectra were recorded at λ_exc_ 340 nm and λ_em_ 380–500. The amount of 10 µM rotenone was used at the end of the experiments to block Complex I and to maximize NAD(P)H content. Standard NADH and NAD(P)H was used for system calibration.

### 2.5. ATP Assay

Intracellular ATP was extracted and measured by HPLC using a two-pump system equipped with a photodiode array detector (Agilent, Santa Clara, CA, USA, 1100 series) following Jones et al. [33] with minor modifications, using a C18 column (Kinetex, Phenomenex, Torrance, CA, USA, 2.6 μm, 250 × 4.6 mm). Nucleotide peaks were identified at λ = 260 nm by comparison and co-elution with the standards. The quantification of different nucleotides was obtained by peak area measurement compared with standard curves.

### 2.6. Measurement of ROS

Oxidative stress was measured in intact cells using the reactive oxygen species indicator 2′,7′-dichlorodihydrofluorescein diacetate (DCFDA, Thermo Fisher Scientific, Waltham, MA, USA), following the method in Bergamini et al. [21] with minor modifications. Briefly, I407 and H9c2 cells were seeded in 96-well plates at 4 × 10^4^ cells/well (Optiplate, Perkin Elmer). After 24 h to allow adhesion, the cells were incubated with different concentrations (ranging from 6.25 nM to 100 nM) of native CoQ10, UBQ or phytosome vehicle dissolved in complete medium for 24 h at 37 °C in 5% CO_2_. After this time, cells were washed with Hank’s balanced salt solution (HBSS) and treated with 10 µM DCFDA (2′,7′-dichlorofluorescein diacetate, DCFH-DA, Thermo Fisher) in DMEM for 30 min. Subsequently, cells were washed with HBSS and treated for 30 min with 130 µM tert-butyl hydroperoxide (TBH) in HBSS. Finally, the cells were washed again with HBSS and the fluorescence value in each well was measured (λexc = 485 nm; λem = 535 nm) with a plate reader (Enspire, Perkin Elmer). Fluorescence emission was normalized on protein content measured by the Lowry method.

### 2.7. Measurement of Mitochondrial Membrane Potential

Cells were cultured in µ-Slide 8 Well (Ibidi, Germany) following the manufacturer instructions and treated with Coenzyme Q as detailed above. Then, cells were stained with the fluorescent carbocyanine dye JC-1 (ThermoFisher Scientific) as reported in [21] with minor modifications. Briefly, cells were incubated with 5 µM JC-1 at 37 °C, 5% CO_2_ for 30 min in culture medium. After this time, the cells were washed twice with Hank’s balanced salt solution (HBSS) and images were acquired using a Nikon C1si confocal microscope (Nikon, Tokyo, Japan). The fluorescence intensity was measured using the ImageJ software standard tool (National Institutes of Health, Bethesda, MD, USA).

### 2.8. Citrate Synthase Assay

Citrate synthase (CS)activity was measured in 100 mM TRIS buffer with 0.1% Triton X-100 at 412 nm (ε = 13,600 M^−1^ cm^−1^) and 30 °C after the addition of 30 μg of cell lysate, 0.1 mM acetyl-CoA, 0.5 mM oxaloacetate and 0.1 mM 5,5′-dithiobis-2-nitrobenzoic acid (DTNB) by using a Jasco-V550 spectrophotometer equipped with a stirring device and thermostatic control.

### 2.9. Ferroptosis Assay

Cells were cultured in 96 wells plates and treated with Coenzyme Q as detailed above. Ferroptosis was induced in incubating cells with different concentrations of RSL3 ((1S,3R)-RSL3), which is an inhibitor of glutathione peroxidase 4 (GPX4) (the CoQ oxidoreductase FSP1 acts parallel to GPX4 to inhibit ferroptosis), or Erastin, which is an inhibitor of the system xc- cystine/glutamate antiporter (FSP-1 is a glutamate independent) for 24 h in complete medium. After this time, cell viability was estimated using a MTT ((3-(4,5-dimethylthiazol-2-yl)-2,5-diphenyltetrazolium bromide) assay. Briefly, the cells were washed with HBSS and incubated for 60 min with 300 μM of MTT in the culture medium. After this time, cells were washed with phosphate buffer and the formazan salts were dissolved in DMSO. The absorbance was measured at 570 nm with a multi-plate reader (EnSpire; PerkinElmer).

### 2.10. Lipid Peroxidation Assay

The determination of lipid peroxidation was performed using the lipid peroxidation sensor dye BODIPY^®^ 581/591 (ThermoFisher Scientific) following the method in Pap et al. [34]. Images were acquired using a Nikon C1si confocal microscope (Nikon, Tokyo, Japan) and fluorescence intensities were quantified using the ImageJ software. Data are reported as red/green fluorescence ratio and the oxidation of the polyunsaturated butadienyl portion of the dye results in a shift of the fluorescence emission peak from ~590 nm to ~510 nm.

### 2.11. Lipid Droplets Assay

Intracellular lipid droplets were stained using the fluorescent dye Nile Red (ThermoFisher scientific) following Greenspan et al. [35] with minor modifications. Lipid droplets quantification was performed using ImageJ software standard tools.

### 2.12. Coenzyme Q10 Internalization Assay

Cells were treated for 30 min with different endocytosis inhibitors: 2 mM amiloride (AM) [36], which inhibits micropinocytosis; 10 µg/mL Chlorpromazine (CPZ) [37], which inhibits clathrin-mediated endocytosis; 200 µM genistein (GN) [38], which inhibits the caveolae-mediated uptake mechanism. After this time, the cells were supplemented for three hours with 100 nM UBQ, 100 nM native CoQ10 and phytosome carrier (Vehicle). Then, cells were carefully washed with a phosphate buffer and CoQ10 was extracted and quantified as described above.

## 3. Results

### 3.1. Coenzyme Q10 Determination in Cells

**Figure 1:**
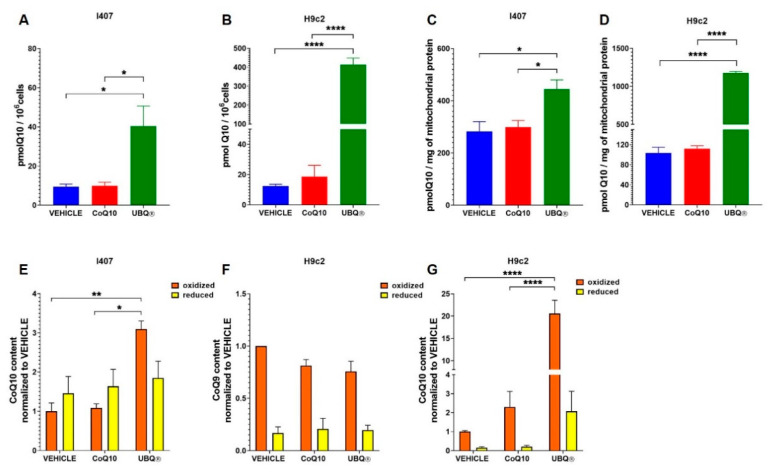
Coenzyme Q10 determination in different cell lines after products incubation. Total CoQ10 content in I407 cells (**A**) and H9c2 cells (**B**) treated for 24 h with 100 nM of CoQ10 phytosome (UBQ), 100 nM of native CoQ10 (Q10) and phytosome (Vehicle) (*n* = 3). Mitochondrial CoQ10 content in I407 cells (**C**) and H9c2 cells (**D**) treated for 24 h with 100 nM UBQ, 100 nM of native Q10 and phytosome (Vehicle) (*n* = 3). Data were normalized to mitochondrial protein content (*n* = 3). (**E**) Reduced and oxidized CoQ10 content in I407 cells treated as above. Data are presented as fold increase over oxidized CoQ10 content of the vehicle-treated sample; (*n* = 3). (**F**) Reduced and oxidized CoQ9 content in H9C2 cells treated as above. (**G**) Reduced and oxidized CoQ10 content in H9C2 cells treated as above. Data are presented as fold increase over oxidized CoQ9 or CoQ10 content of the vehicle-treated sample; (*n* = 3). For all panels, data are presented as the mean ± s.e.m, * *p* ≤ 0.05; ** *p* ≤ 0.01; **** *p* ≤0.0001. Statistical analysis was performed using one-way ANOVA followed by Tukey’s honest significant difference test.

To study the bioavailability of a CoQ10 phytosome formulation (UBQ), we supplemented two cell lines (H9c2 and I407) with 100 nM of UBQ, native CoQ10 and with phytosome alone as control (vehicle). After 24 h of treatment, we quantified the CoQ10 levels by HPLC. Figure 1A,B reported the total cellular level of CoQ10 in I407 and H9c2, respectively, showing a dramatic increase in ubiquinone levels in the UBQ treated cells, which is particularly evident in H9c2 cell line. In I407 cells, the UBQ incubation significantly increased the CoQ_10_ content from 0.01 nmoles/10^6^ cells to 0.05 nmoles/10^6^ cells. In H9c2 cells the UBQ incubation significantly increased the CoQ_10_ content from 0.013 nmoles/10^6^ cells to 0.41 nmoles/10^6^ cells. On the other hand, in both cell lines, the incubation with CoQ10 was not able to significantly increase the ubiquinone level in comparison to the control. Moreover, we isolated the mitochondria from cells after products incubation and found that the UBQ treatment increased the mitochondrial ubiquinone level than when compared with CoQ10 treated cells and vehicle treated controls (Figure 1C,D). In mitochondria isolated from I407 cells, the UBQ incubation significantly increased the CoQ_10_ content from 0.28 nmoles/mg to 0.44 nmoles/mg. In mitochondria isolated from H9c2 cells, the UBQ incubation significantly increased the CoQ_10_ content from 0.10 nmoles/10^6^ cells to 1.18 nmoles/10^6^ cells.

In order to further investigate the effect of the phytosome formulation on CoQ10 bioavailability, we determined the redox state of CoQ10 in cultured cells after supplementation with UBQ, native CoQ10 and the vehicle. As reported in Figure 1E, we found that UBQ increased by three times the oxidized ubiquinone levels in I407 cells without affecting the levels of the reduced form, whereas the supplementation with CoQ10 had no effect on oxidized or reduced CoQ10 levels. In H9c2 cells, the UBQ treatment dramatically increased by 20 times the oxidized CoQ10 content, with a concomitant increase in the reduced form by 13 times (Figure 1G). Since H9c2 is a rat cell line, the endogenous ubiquinone is mainly present as a CoQ9 analogue; the supplementation with UBQ, CoQ10 or the vehicle did not change the level and the redox state of CoQ9 (Figure 1F).

### 3.2. Bioenergetic Effects of UBQ Supplementation

**Figure 2:**
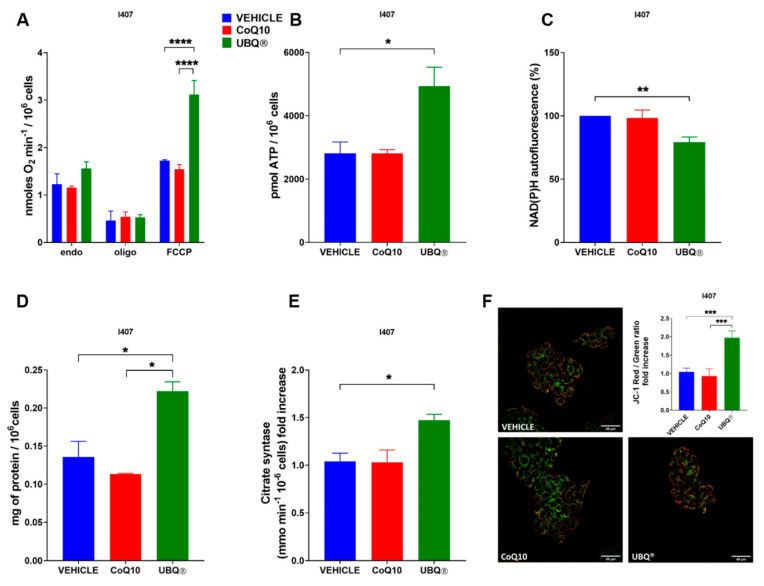
Bioenergetic effects of UBQ treatment in I407 cells. Cells were incubated with 100 nM CoQ10 phytosome (UBQ), native CoQ10 (Q10) and phytosome (vehicle) as described in the materials and methods section. (**A**) Mitochondrial Oxygen consumption rate in intact cells (endogenous respiration, endo), in the presence of 1 µM oligomycin A (oligo) and 0.25–1 µM carbonyl cyanide 4-(trifluoromethoxy) phenylhydrazone (FCCP). Data are expressed as nanomoles of oxygen per minute and normalized to the cell number (*n* = 3). (**B**) Total cellular ATP quantification in cell lysate (*n* = 3). (**C**) NAD(P)H autofluorescence determination in intact cells. Data were normalized to cell number and expressed as a percentage of vehicle-treated sample fluorescence signal; (*n* = 3). (**D**) Cellular protein content determination (*n* = 3). (**E**) Citrate synthase activity determination normalized to cell number. Data are shown as fold increase over vehicle-treated sample; (*n* = 3). (**F**) Representative micrographs of intact cells stained with the fluorescent dye JC-1. Quantitative analysis of Red/Green fluorescence ratio was performed using ImageJ software. For each condition, at least two randomly chosen fields were analyzed. Data are presented as Red/Green fluorescence ratio fold increase over the vehicle-treated sample; (*n* = 5). For all panels, data are presented as the mean ± s.e.m, * *p* ≤ 0.05; ** *p* ≤ 0.01; *** *p* ≤ 0.001; **** *p* ≤ 0.0001. Statistical analysis was performed using one-way ANOVA followed by Tukey’s honest significant difference test.

**Figure 3:**
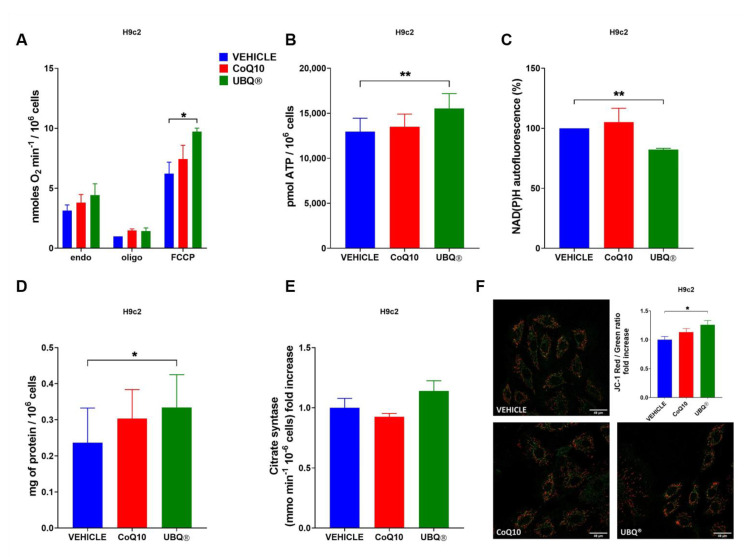
Bioenergetic effects of UBQ treatment in H9c2 cells. Cells were incubated with 100 nM CoQ10 phytosome (UBQ), native CoQ10 (Q10) and phytosome (vehicle) as described in the materials and methods section. (**A**) Mitochondrial Oxygen consumption rate in intact cells (endogenous respiration, endo) in the presence of 1µM oligomycin A (oligo) and 0.25–1 µM carbonyl cyanide 4-(trifluoromethoxy) phenylhydrazone (FCCP). Data are expressed as nanomoles of oxygen per minute and normalized to cell number; (*n* = 3). (**B**) Total cellular ATP quantification in cell lysate; (*n* = 3). (**C**) NAD(P)H autofluorescence determination in intact cells. Data were normalized to cell number and expressed as a percentage of vehicle-treated sample fluorescence signal; (*n* = 3). (**D**) Total protein content determination normalized to cell number; (*n* = 3). (**E**) Citrate synthase activity determination normalized to cell number. Data are shown as fold increase over the vehicle-treated sample; (*n* = 3). (**F**) Representative micrographs of intact cells stained with the fluorescent dye JC-1. For each condition, at least three fields were randomly selected. Quantitative analysis of Red/Green fluorescence ratio was performed using the ImageJ software. Data are presented as Red/Green fluorescence ratio fold increase over the vehicle-treated sample; (*n* = 5). For all panels, data are presented as the mean ± s.e.m, * *p* ≤ 0.05; ** *p* ≤ 0.01. Statistical analysis was performed using one-way ANOVA followed by Tukey’s honest significant difference test.

We investigated the effect of UBQ and native CoQ10 supplementation in H9c2 and I407 cells on different bioenergetic parameters, such as oxygen consumption, ATP and NADH level, protein content, transmembrane potential and citrate synthase activity. We determined the oxygen consumption rate in intact cells using polarographic techniques in basal condition, in the presence of the ATPase inhibitor oligomycin A and in the presence of the uncoupler carbonyl cyanide 4-(trifluoromethoxy) phenylhydrazone (FCCP). We showed that UBQ was able to significantly increase the uncoupled rate of oxygen consumption both in I407 (1.55 nmoles O_2_ min^−1^/10^6^ cells in the controls; 3.12 nmoles O_2_ min^−1^/10^6^ cells in UBQ) and in H9c2 cells (4.44 nmoles O_2_ min^−1^/10^6^ cells in the controls; 9.72 nmoles O_2_ min^−1^/10^6^ cells in UBQ), while the CoQ10 treatment did not affect it (Figure 2A and Figure 3A).

Since the ubiquinone plays a fundamental role in the formation of the mitochondrial transmembrane potential (ΔΨm), we measured the ΔΨm in intact cells using the fluorescent dyes JC-1 after supplementation with the vehicle, UBQ and CoQ10. Figure 2F and Figure 3F showed that UBQ treatment significantly increased the ΔΨm in both I407 (1.97 fold increase) and H9c2 (1.26 fold increase) cell lines.

Moreover, the cellular ATP content (Figure 2B and Figure 3B) was measured and it was found that in I407 the UBQ treatment increased the ATP level by some 70% in comparison with the vehicle, while in H9c2 the ATP increased was less marked albeit significantly different from the vehicle treated controls. Since active mitochondria oxidizes NADH to sustain the oxidative phosphorylation activity, we assessed its level by fluorometric analysis in I407 and H9c2 cells supplemented with UBQ and native CoQ10. Figure 2C and Figure 3C showed that UBQ treatment significantly decreased the NADH level in comparison to vehicle treatment in I407 (−20.8%) and H9c2 (−17.7%) cells, respectively. Moreover, we measured the cellular protein content by using the Lowry method. We found that in I407 cells treated with UBQ, the ratio between protein content and cell number significantly increased in comparison with CoQ10 and vehicle-treated cells (Figure 2D). Similarly, in H9c2 cells treated with UBQ, the ratio between protein content and cell number significantly increased in comparison with CoQ10 and vehicle-treated cells (Figure 3D).

Finally, we assessed the effect of ubiquinone supplementation on cellular mitochondrial content by measuring the activity of the citrate synthase (CS) enzyme. Figure 3E showed that UBQ increased the CS activity in I407 (1.42-fold increase), suggesting an increased mitochondrial biogenesis. It is important to note that the treatment with CoQ10 had no significant effects on CS activity.

### 3.3. CoQ10 Phytosome Protects Cells from Oxidative Stress

**Figure 4:**
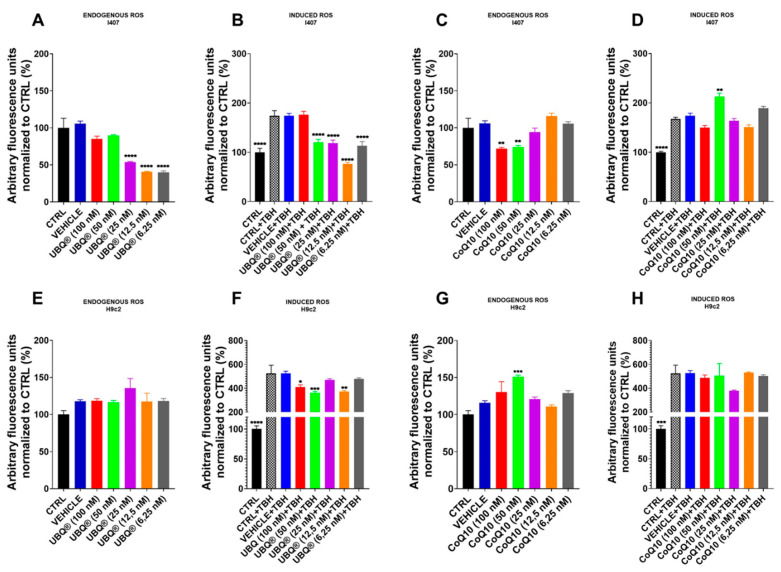
Oxidative stress determination in I407 and H9c2 cells. Cells were incubated with CoQ10 phytosome (UBQ), native CoQ10 (Q10) and phytosome (vehicle) at different concentrations for 24 h. Endogenous oxidative stress was measured in intact I407 (**A**,**C**) and H9c2 (**E**,**G**) cells using the fluorescent probe 2′,7′-dichlorodihydrofluorescein diacetate (DCFDA). Oxidative stress was induced in intact I407 (**B**,**D**) and H9c2 (**F**,**H**) cells using 130 µM of tert-butyl hydroperoxide (TBH). Data were normalized to protein content and presented as a percentage of untreated control fluorescence signal (*n* = 48). For all panels, data are presented as the mean ± s.e.m, * *p* ≤ 0.05; ** *p* ≤ 0.01; *** *p* ≤ 0.001; **** *p* ≤ 0.0001. Statistical analysis was performed using one-way ANOVA followed by Tukey’s honest significant difference test.

Reduced CoQ10 is a lipid-soluble antioxidant, which protects the membranes from peroxidation. In order to assess the protective effect of UBQ and CoQ10 on endogenous and induced oxidative stress, we treated I407 and H9c2 cells with different concentrations (ranging from 6.25 to 100 nM) of UBQ and native CoQ10 for 24 h. After this period, we measured the cellular oxidative stress using the fluorogenic probe DCFDA, which is a cell-permeant reactive oxygen species indicator. We evaluated the oxidative stress in standard culture conditions (endogenous ROS) after 30 min of exposure to the organic peroxide TBH. The results reported in Figure 4 showed that the treatment with low doses of UBQ (12.5 nM and 6.25 nM) was more effective in counteracting the oxidative stress both in endogenous and TBH-induced conditions. Furthermore, the antioxidant effect of UBQ treatment was higher in I407 than in H9c2 cells, while the treatment with native CoQ10 was almost ineffective in both cell lines. These results confirm the hypothesis that an excessive amount of oxidized CoQ10 in cells might be detrimental to its antioxidant effect.

### 3.4. CoQ10 Phytosome Protects Cells from Lipid Membrane Peroxidation

**Figure 5:**
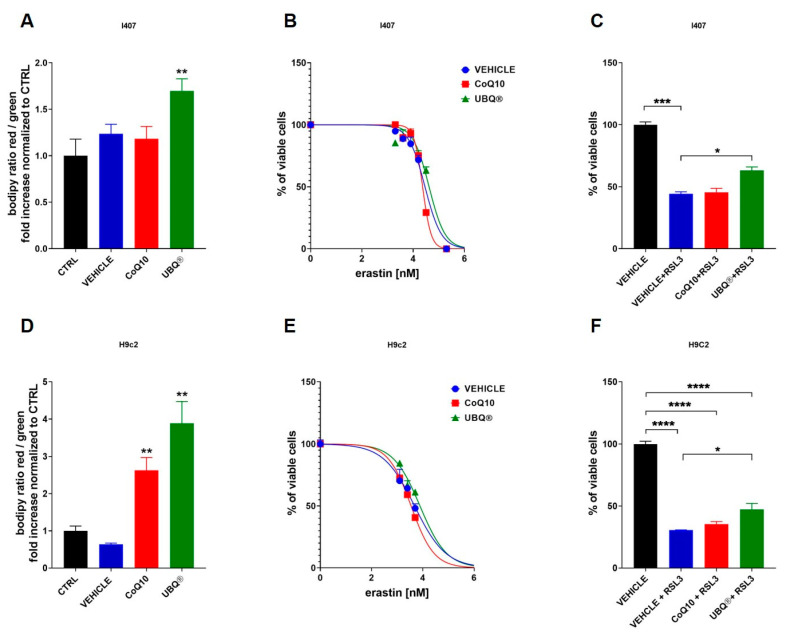
Lipid membrane peroxidation evaluation in I407 and H9c2. Cells were treated with 100 nM CoQ10 phytosome (UBQ), native CoQ10 (Q10) and phytosome (vehicle) or untreated as control. The peroxidation status of membrane lipids was determined using the fluorescent dye BODIPY™ 581/591 C11 in fixed I407 (**A**) and H9c2 (**D**) cells by confocal microscopy. For each condition, at least three fields were randomly selected. Quantitative analysis of Red/Green fluorescence ratio was performed using the ImageJ software. Data are presented as fold increase over untreated controls (*n* = 5). The viability of I407 (**B**) and H9c2 (**E**) cells in the presence of different concentrations of the ferroptosis-inducer erastin was assessed by MTT assay after 24 h of incubation (*n* = 3). The viability of I407 (**C**) and H9c2 (**F**) cells after 24 h of incubation with 1 μM and 300 nM, respectively, of the GPX4 inhibitor RSL3 was assessed by MTT assay (*n* = 3). Data are presented as a percentage of vehicle-treated viable cells. For all panels, data are presented as the mean ± s.e.m, * *p* ≤ 0.05; ** *p* ≤ 0.01; *** *p* ≤ 0.001; **** *p* ≤ 0.0001. Statistical analysis was performed using one-way ANOVA followed by Tukey’s honest significant difference test.

In order to study the protective effect of CoQ10 on lipid membrane peroxidation, the cells were stained with the lipid peroxidation sensor BODIPY™ 581/591 C11. The oxidation of the polyunsaturated butadienyl portion of the dye results in a shift of the fluorescence emission peak from ~590 nm to ~510 nm and can be used to measure antioxidant activity in lipid environments by exploiting its decrease in red/green fluorescence ratio upon interaction with peroxyl radicals. Figure 5A,D showed that UBQ treatment significantly protected the membrane lipids of I407 and H9c2 cells from peroxidation.

On the other hand, the native CoQ10 treatment significantly increased the red/green ratio of BODIPY dye only in H9c2 cells.

In order to further investigate the antioxidant activity of UBQ at the membrane level, lipid peroxidation was induced by using two inhibitors of intracellular reduced glutathione level (GSH): erastin, which inhibits the cysteine import, and RSL3, which inhibits the GPX4 enzyme. Figure 5B,E showed the percentage of viable I407 and H9c2 cells in the presence of a different concentration of erastin, determined by MTT assay. Results showed that the UBQ supplementation in I407 increased the IC_50_ of erastin from 29 μM (vehicle) to 42 μM; similarly, in H9c2 cells the UBQ treatment increased the IC_50_ of erastin from 4.7 µM (vehicle) to 7.7 µM. Native CoQ10 supplementation in I407 non-significantly decreased the IC50 of erastin from 29 μM (vehicle) to 23 μM; similarly, in H9c2 cells, the native CoQ10 treatment non-significantly decreased the IC50 of erastin from 4.7 µM (vehicle) to 3.5 µM. These data suggest that UBQ treatment can protect cells from oxidative stress in the presence of low GSH levels.

In order to confirm this latter point, we treated the cells with the specific ferroptosis activator RSL3 (Figure 5C,F) and found that UBQ treatment was able to improve cell viability in both I407 (44.2% of viable cells in the presence of Vehicle + RSL3 vs. 63.1% of viable cells in the presence of UBQ + RSL3) and in H9c2 (30.5% of viable cells in the presence of Vehicle + RSL3 vs. 47.28% of viable cells in the presence of UBQ + RSL3). The treatment with 100nM native CoQ10 did not protect both I407 and H9c2 cells from RSL3 induced ferroptosis.

### 3.5. Cellular Uptake of CoQ Phytosome Formulation

**Figure 6:**
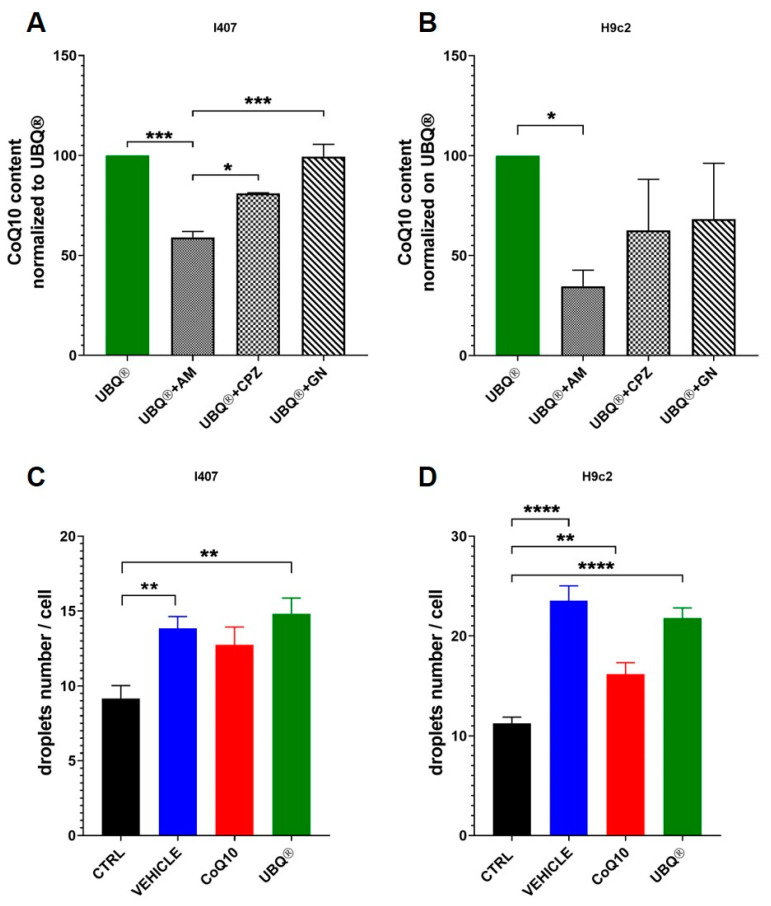
Cellular uptake of UBQ formulation. I407 (**A**) and H9c2 (**B**) cells were incubated for 30 min with 2 μM of amiloride (AM), 10 μg/mL chlorpromazine (CPZ) and 200 μM genistein (GN) and then further incubated for three hours with 100 nM of CoQ10 phytosome formulation (UBQ). The Coenzyme Q10 content was quantified by HPLC. Data are presented as a percentage of cellular Coenzyme Q10 content compared to control samples treated with UBQ. (*n* = 3). The presence of lipid droplets after products incubation was detected in fixed I407 (**C**) and H9c2 (**D**) cells by confocal microscopy using the fluorescent dye Nile Red. For each condition, at least three fields were randomly selected and the quantification of the number of positive Nile red spots per single cell was performed using the ImageJ software. For all panels, data are the mean ± s.e.m, * *p* ≤ 0.05; ** *p* ≤ 0.01; *** *p* ≤ 0.001; **** *p* ≤ 0.05. Statistical analysis was performed using one-way ANOVA followed by Tukey’s honest significant difference test.

In order to study the mechanism of internalization of the CoQ phytosome formulation, we treated I407 and H9c2 cells with three endocytosis inhibitors: amiloride (AM), which inhibits micropinocytosis; Chlorpromazine (CPZ), which inhibits clathrin-mediated endocytosis; genistein (GN), which inhibits the caveolae-mediated uptake mechanism. After exposure to endocytosis inhibitors, the cells were incubated with 100 nM of UBQ and the CoQ10 levels were assayed by HPLC after three hours. Figure 6A, B showed that the uptake of UBQ formulation was sensitive to AM inhibition in both I407 and H9C2 cells. The AM treatment decreased the UBQ uptake by 40% in I407 and by 65.35% in H9C2 cells. Moreover, we investigated the presence of intracellular lipid droplets after CoQ supplementation using the fluorescent dye Nile red, which stains the neutral lipids within cells. Figure 6C, D showed that the number of lipid droplets for cells increased dramatically after UBQ, CoQ10 and vehicle treatment, which suggests that both CoQ10 and lipids from phytosome are, at least in part, internalized as lipid aggregates.

## 4. Discussion

Coenzyme Q10 (CoQ10, ubiquinone) is a redox-active lipid present across all domains of life. It is mainly found in the mitochondrial inner membrane, where it is an obligate component of the electron transport chain and thus essential for the oxidative phosphorylation process. Its high concentration in tissues with elevated energy demands, such as the heart, skeletal muscle and neurons, highlights its bioenergetic role. Moreover, CoQ10 is the only lipid-soluble antioxidant endogenously synthesized. It is essential to protect membrane components against oxidations and to prevent oxidative-stress-dependent cellular damage [39,40]. The tissue concentration of CoQ10 declines during ageing and in the presence of oxidative stress; this constitutes the basis on which the clinical use of CoQ10 is most recommended [41,42].

CoQ10 was used for the first time in 1973 to treat patients with congestive heart failure and, after that, the clinical interest in CoQ10 supplementation increased over the years. Currently, it is the third most consumed nutritional supplement after fish oil and multivitamins [43]. CoQ10 is a very hydrophobic molecule with high molecular weight: These chemical characteristics make it highly water-insoluble and affects its bioavailability; for these reasons, its intestinal adsorption results are incomplete and slow. Several toxicological studies demonstrated that CoQ10 supplementation does not cause adverse effects, nor does it influence endogenous CoQ10 biosynthesis, as extensively discussed in a comprehensive review [44]. Bioavailability studies have shown that there is no linear correlation between the amount of CoQ10 supplemented and its plasma concentration; in particular, it has been reported that the higher the supplemented dose, the lower the percentage of the dose absorbed due to the achievement of a maximum plateau level of CoQ10, after which its absorption is reduced [45].

Currently, several formulations of CoQ10 have been developed and studied to overcome the limited bioavailability of the molecule. In this work, we analyzed the cellular uptake, distribution and the bioenergetic effects of a highly bioavailable food-grade lipid-based formulation of CoQ10 (UBQ or UBIQSOME: a CoQ10 dispersion in a lecithin matrix [23]) in comparison with standard CoQ10. UBQ has been recently reported to have an improved solubility and a good bioavailability in human plasma and muscle tissues [23,24,25]. These evidences suggests an investigation in deep the cellular mechanisms in order to better understand the efficiency of the observed gain in CoQ10 absorption. As cellular models, we used a human epithelial intestine cell line (I407) and a rat embryonic myoblast cell line (H9c2). Our results showed that UBQ induced a significant increase in the cellular and mitochondrial CoQ10 content. It is important to note that while the uptake of standard CoQ10 is similar in the two cell lines tested, the CoQ10 phytosome dispersion is absorbed differently by these cells, suggesting that CoQ10 formulation might have a significant impact on cell-specific uptake of ubiquinone.

Since only the reduced form of CoQ10 has antioxidant properties [46], we measured the levels of both reduced (CoQ_red_) and oxidized (CoQ_ox_) forms of CoQ10 in our cell lines. Control H9c2 cells exhibited a preponderance of CoQ_ox_ form over CoQ_red_, while in I407 cells the levels of both forms were comparable. In I407, treatment with UBQ equally increased both CoQ10 forms and protected cells from both endogenous and TBH-induced oxidative stress. On the other hand, in H9c2 cells, supplementation with UBQ considerably increased the CoQ_ox_ level over the CoQ_red_ level. The imbalance in the CoQox/CoQred forms ratio could represent the rationale of why UBQ supplementation in H9c2 cells offers modest protectection against TBH-induced oxidative stress and is not effective against endogenous oxidative stress. The antioxidant effect of CoQ10 appears closely related to the ability of cells to efficiently reduce it rather than their ability to internalize it in high doses; present data show that not only does an excessive concentration of oxidized CoQ10, as the native form is not formulated, not protect against oxidative stress but it may also have a slight pro-oxidant effect [21,47]. These latter observations highlight that a proper CoQ10 formulation, which increases the bioavailability allowing incubation at lower dosages [48], is essential to avoid a non-specific intracellular accumulation.

Further investigation the antioxidant properties of CoQ10 showed that UBQ protected cell membranes from lipid peroxidation. Notably, UBQ treatment protected both cell lines from ferroptosis, a type of cell death related to several neurological diseases [49,50,51].

UBQ decreased oxidative stress and improved several bioenergetic parameters in H9c2 and I407 cells. These effects are particularly relevant, taking into consideration that mitochondria are also involved in the pathogenesis and progression of several diseases, including cancer and neurodegenerative and cardiovascular disorders. The respiration data showed that UBQ significantly improved the FCCP/oligomycin A oxygen consumption ratio in both cell lines and this indicates an increased spare capacity to sustain energy requirement. Moreover, UBQ improved the cellular ATP and protein content as well as the mitochondrial transmembrane potential. The literature reported that the intracellular concentration on NAD(P)H increases in the presence of hypoxia [52] or decreased OXPHOS [53]. Accordingly, UBQ decreased the cellular NAD(P)H level in comparison to the control, indicating that the treatment increased the oxidative metabolism. Furthermore, Noh et al. [54] reported that CoQ10 protected ONH astrocytes from oxidative stress by triggering mitochondrial biogenesis, which possibly by modulates the expression of mitofilin and peroxisome proliferator-activated receptor-ɣ coactivator-1 protein. Accordingly, in I407 cells UBQ significantly increased citrate synthase activity, which is a recognized mitochondrial mass marker [55]. It is important to note that the treatment with native CoQ10 has not been effective in improving any of the bioenergetic parameters and this highlights the importance of the formulation in the use of CoQ10. We sought to investigate the mechanism of UBQ internalization in our cell models using different endocytosis inhibitors. We found that the macropinocytosis inhibitor amiloride was the most effective in blocking CoQ10 entry. Macropinocytosis is characterized by the nonspecific internalization of large amounts of extracellular fluid, solutes and membranes into large endocytic vesicles known as macropinosomes [56]; it is conceivable that cells may exploit the process of macropinocytosis to internalize the CoQ10 phytosome formulation [57]. The presence of cytoplasmic lipid droplet after UBQ treatment was detected using the Nile Red dye. Both the treatment with the phytosome vehicle and UBQ significantly increased the number of droplets and this suggests a laborious intracellular distribution due to the extremely lipophilic nature of the molecule. In vivo, the tissue distribution of CoQ10 is still a matter of debate [45,58]. Oral supplementation is always efficient in increasing plasma CoQ10 levels, while the results of CoQ10 distribution in tissues are often controversial. Remarkably, two studies reported a slight muscular accumulation of CoQ10 after treatment with UBQ in healthy aging athletes [24,25]. Bentinger et al., using a [^3^H]CoQ10, showed that its uptake was high in liver, spleen and white blood cells and very low in muscle, kidney and the brain [59]. These authors found that CoQ10 accumulated mainly in lysosomes and only in a small amount in mitochondria; they concluded that this specific subcellular distribution of CoQ10 might be functionally favorable for the cells, especially considering the particular function of CoQ10 in these subcellular compartments.

Our results suggest that even in a cellular model, CoQ10 supplementation induced a different intracellular distribution, favoring CoQ10 accumulation in lipid droplets rather than in mitochondria. Interestingly, we found that the two cell lines used in this work showed a differential CoQ10 uptake: lower for the intestinal epithelial cell line (I407) and higher for the embryonal cardiac cell line (H9c2), which confirms that a limiting step in CoQ10 absorption could be represented by the intestinal uptake and this is in favor of the phytosome formulation with respect to the native CoQ10.

## 5. Conclusions

In conclusion, our data show that the food-grade formulation UBQ, by increasing cellular and mitochondrial CoQ10 content, improved cellular bioenergetic parameters in in vitro models. We revealed that the antioxidant effect of CoQ10 is closely related to an efficient formulation that may finally result in a correct CoQ10 intracellular distribution and result in the ability of the cell to efficiently reduce it rather than to its total concentration. Finally, we found that the mechanism of macropinocytosis underlies the cellular internalization of the phytosome formulation.

In this scenario, the use of a suitable CoQ10 formulation is essential for improving bioenergetic parameters and limiting the nonspecific cellular accumulation of CoQ10 due to high-dose supplementation.

## Data Availability

Data is contained within the article.
